# Retraction: Decoding the Past, Informing the Future: Artificial Intelligence as a Bridge Between Ancient Egyptian Medical Knowledge and Modern Surgical Practice 

**DOI:** 10.7759/cureus.r241

**Published:** 2026-09-09

**Authors:** Zain Khalpey, Hossam Amer, Alyssa Abraham

**Affiliations:** 1 Cardiothoracic Surgery, HonorHealth, Scottsdale, USA; 2 Cardiac Research, Khalpey Artificial Intelligence (AI) Lab, Scottsdale, USA; 3 Cardiothoracic Surgery, Khalpey Artificial Intelligence (AI) Lab, Scottsdale, USA

This article has been retracted by the Editor-in-Chief. Following concerns raised by a reader, a post-publication review by the journal found that the study counts reported in the Results (fifteen machine learning studies and eleven imaging studies) correspond to no sources in the reference list, that several references could not be located as printed, and that several others do not support the statements citing them. The authors were unable to substantiate the reported study counts. The authors confirmed the factual findings set out above and proposed corrections. They disagree with the decision to retract rather than correct the articles.

